# Development and Utility of Practical Indicators of Critical Outcomes in Dengue Patients Presenting to Hospital: A Retrospective Cross-Sectional Study

**DOI:** 10.3390/tropicalmed8040188

**Published:** 2023-03-25

**Authors:** Chia-Yu Chi, Tzu-Ching Sung, Ko Chang, Yu-Wen Chien, Hsiang-Chin Hsu, Yi-Fang Tu, Yi-Ting Huang, Hsin-I Shih

**Affiliations:** 1 National Mosquito-Borne Diseases Control Research Center, National Health Research Institutes, Miaoli County 35053, Taiwan; 2 National Institute of Infectious Diseases and Vaccinology, National Health Research Institutes, Miaoli County 35053, Taiwan; 3 Department of Microbiology & Immunology, College of Medicine, National Cheng Kung University, Tainan 701401, Taiwan; 4 School of Medicine for International Students, College of Medicine, I-Shou University, Kaohsiung 840203, Taiwan; 5 Department of Internal Medicine, School of Medicine, College of Medicine, Kaohsiung Medical University, Kaohsiung 807378, Taiwan; 6 Division of Infectious Diseases, Department of Internal Medicine, Kaohsiung Municipal Siaogang Hospital, Kaohsiung 81267, Taiwan; 7 Department of Occupational and Environmental Medicine, National Cheng Kung University Hospital, College of Medicine, National Cheng Kung University, Tainan 704302, Taiwan; 8 School of Medicine, College of Medicine, National Cheng Kung University, Tainan 701401, Taiwan; 9 Department of Pediatrics, National Cheng Kung University Hospital, College of Medicine, National Cheng Kung University, Tainan 704302, Taiwan; 10 Department of Emergency Medicine, National Cheng Kung University Hospital, College of Medicine, National Cheng Kung University, Tainan 704302, Taiwan; 11 Department of Public Health, College of Medicine, National Cheng Kung University, Tainan 701401, Taiwan

**Keywords:** dengue, severe dengue, critical dengue

## Abstract

Global travel and climate change have drastically increased the number of countries with endemic or epidemic dengue. The largest dengue outbreak in Taiwan, with 43,419 cases and 228 deaths, occurred in 2015. Practical and cost-effective tools for early prediction of clinical outcomes in dengue patients, especially the elderly, are limited. This study identified the clinical profile and prognostic indicators of critical outcomes in dengue patients on the basis of clinical parameters and comorbidities. A retrospective cross-sectional study was conducted in a tertiary hospital from 1 July 2015 to 30 November 2015. Patients diagnosed with dengue were enrolled, and the initial clinical presentations, diagnostic laboratory data, details of the underlying comorbidities, and initial management recommendations based on 2009 World Health Organization (WHO) guidelines were used to evaluate prognostic indicators of critical outcomes in dengue patients. Dengue patients from another regional hospital were used to evaluate accuracy. A group B (4 points) classification, temperature < 38.5 °C (1 point), lower diastolic blood pressure (1 point), prolonged activated partial thromboplastin time (aPTT) (2 points), and elevated liver enzymes (1 point) were included in the scoring system. The area under the receiver operating characteristic curve of the clinical model was 0.933 (95% confidence interval [CI]: 0.905–0.960). The tool had good predictive value and clinical applicability for identifying patients with critical outcomes.

## 1. Introduction

Dengue is the most rapidly spreading mosquito-borne viral disease in the world, with approximately 50 million dengue infections occurring annually and 4 billion people at risk of dengue [1]. The number of countries with endemic or epidemic dengue has increased drastically in recent years as a consequence of the spread of dengue viruses due to frequent global travel and climate change [2]. There have been cases of dengue fever in southern Taiwan since 1987 [3]. After a decade with few dengue cases, the largest outbreak occurred in 2015. This outbreak, caused by dengue serotype 2, led to a total of 43,419 cases and 228 deaths [4]. The highest prevalence rate was observed among patients aged over 70 years [5], which was in contrast to the predominantly younger age of dengue patients in other Southeast Asian countries [6,7,8].

Dengue is a dynamic systemic disease with a wide spectrum of clinical presentations. Most patients recover after a self-limiting nonsevere clinical course; however, a small proportion of infections progress to severe disease, mostly characterized by plasma leakage with or without hemorrhage [9]. Early recognition and appropriate treatment of dengue may prevent patients from developing more severe clinical conditions and reduce the case fatality rate [10]. To help clinicians decide when a patient should be monitored and how intensively a patient should be treated, the WHO released the 2009 classification system, which divides patients with symptomatic dengue virus infections into patients with or without warning signs and patients with severe dengue. Accordingly, case management focuses on three categories (groups A, B, and C). However, as indicated in the management recommendations, even dengue patients without warning signs who are discharged from the hospital can develop critical outcomes. The sensitivity of the WHO 2009 classification criteria for identifying patients who need intensive care at the first presentation has been shown to be 52% [11]. The sensitivity is even lower in older patients, resulting in substantial challenges regarding the achievement of an early clinical diagnosis in older patients [12]. Management at the primary and secondary care levels, where patients are first seen and evaluated, is critical for determining the clinical outcome of dengue. A well-managed frontline response not only reduces the number of unnecessary hospital admissions but also saves the lives of dengue patients. Simple but effective tools can help identify those at risk of developing critical outcomes who need intensive care.

Information on the practicality and cost-effectiveness of tools for the early prediction of critical outcomes in dengue patients, especially older patients, has been limited. Previous studies have identified the risk factors associated with intensive care unit (ICU) admission or mortality due to dengue [11,13,14,15,16]. Most of these studies were limited by small sample sizes. A number of identified risk factors for critical outcomes in dengue patients only develop as the disease progresses to the critical phase and are not observable in the early stage. In addition, patients with critical dengue experience a cytokine storm with high levels of circulating proinflammatory cytokines, leading to endothelial activation and vascular leakage with hemorrhage and shock [17,18]. These cytokine levels, however, can only be measured in research laboratories and not in clinical settings.

Recognizing the importance of the early identification of dengue patients at risk for critical outcomes, we developed and externally evaluated a simple scoring tool that can be easily, inexpensively, and usefully applied in clinical settings to identify dengue patients in the early phase of the disease who are at greater risk of critical outcomes.

## 2. Materials and Methods

### 2.1. Study Design and Setting

According to the Communicable Disease Control Act in Taiwan, dengue fever is classified as a category 2 notifiable communicable disease that should be reported to the Taiwan Center for Disease Control (CDC) within 24 h. Confirmed dengue patients need to meet the clinical (any of the two inclusive symptoms including headache/back pain/muscle pain/arthralgia/bone headache, rash, leukopenia, nausea/vomiting, positive tourniquet test, or any of the warning signs), laboratory (positive dengue virus isolated from blood, positive molecular nucleotides from clinical specimens, positive nonstructural protein (NS1) in serology, positive IgM or IgG in serology in the acute dengue stage, positive IgM or IgG conversion or fourfold elevation in paired serology), and epidemiological (positive dengue patients are nearby or travel history to dengue-affected areas) criteria [19,20].

This retrospective cross-sectional study was conducted using hospital-based clinical data from 1 July 2015 to 30 November 2015 associated with the dengue outbreak of 2015. Clinical data on dengue adult patients (≥18 years) were collected from dengue cases reported to Taiwan CDC in National Cheng Kung University Hospital, a 1000-bed tertiary care teaching and major dengue-designated treatment hospital in Tainan, southern Taiwan. All the enrolled patients for analysis included those with dengue diagnosis and presentation that met the Taiwan CDC’s notifiable infectious disease criteria. Patients visiting either the emergency department (ED) or outpatient department (OPD) were reviewed.

The clinical information included initial clinical symptoms, temperature, respiratory rate, pulse, and blood pressure, and the laboratory data we used were either in the initial blood sample taken within 2 h after the ED triage or during the OPD visit. The diagnosis of dengue was confirmed by serum NS1 detection (Standard Diagnostics, Inc., Kyonggi-do, Republic of Korea) [21], enzyme-linked immunosorbent assay (ELISA)-based detection of specific IgM antibodies, or real-time polymerase chain reaction (RT–PCR) (Lightmix^®^ kit, TIB MOLBIOL GmbH, Berlin, Germany) based on the Taiwan CDC’s criteria. Patient data, including demographic characteristics, triage parameters, vital signs, clinical symptoms and signs, laboratory data, diagnoses, and outcomes (i.e., critical or noncritical status), were collected from electronic medical records. Symptoms such as abdominal pain and vomiting several times in one day or that lasted for more than one day were recorded in the medical records. We analyzed the information that was presented in the first three days for further analysis.

Adult dengue patients diagnosed in another major designated dengue treatment hospital outfitted with 400 beds, 20 of which were ICU beds, in another dengue-endemic region during 2011–2015 were used for external utility evaluation of the scoring system. All criteria used for the external utility evaluation were the same as those used in the study hospital.

### 2.2. Definition of Cases and Variables

**Figure 1:**
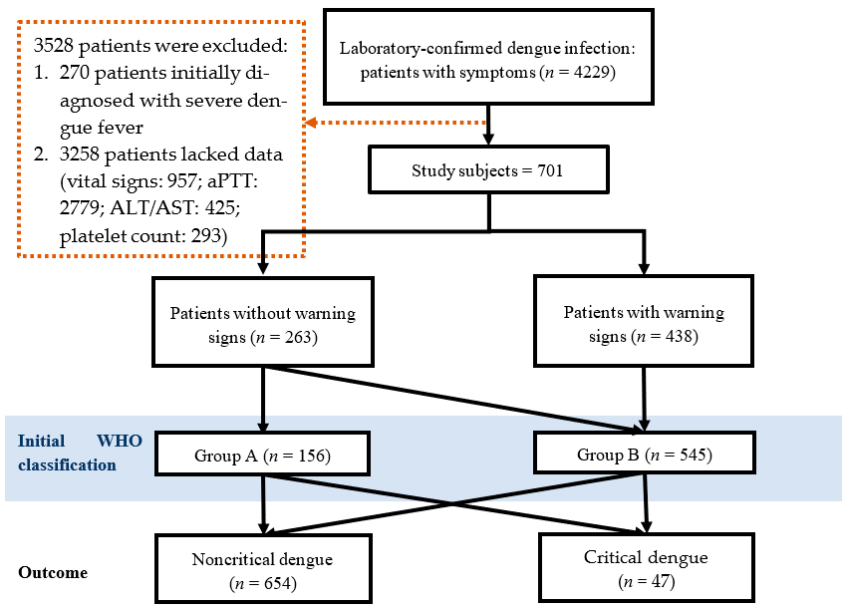
Flow chart of the patients who met the inclusion/exclusion criteria for the study population.

Figure 1 shows the flowchart of the study. A total of 4229 symptomatic laboratory-confirmed dengue patients were enrolled initially. However, 3528 dengue patients were excluded from further analysis, including 270 dengue patients initially diagnosed with severe dengue and 3258 dengue patients who lacked complete vital signs and clinical data, e.g., activated partial thromboplastin time (aPTT) and alanine transaminase (ALT) and aspartate transaminase (AST) or platelet levels. A total of 701 dengue patients were classified on the basis of presentation at the first hospital visit as groups A (may be sent home) and B (referred for in-hospital care) according to the 2009 WHO criteria. Group A was defined as patients who did not have warning signs and who were able to tolerate adequate volumes of oral fluids and pass urine at least once every 6 h. Group B was defined as patients with any of the following features: (1) coexisting conditions, such as pregnancy, infancy, old age, diabetes mellitus (DM), renal failure, or chronic hemolytic diseases; (2) social circumstances, such as living alone or living far from the hospital; or (3) existing warning signs (abdominal pain or tenderness, persistent vomiting, clinical fluid accumulation, mucosal bleeding, lethargy/restlessness, liver enlargement (>2 cm), or elevated hematocrit level). Group C was defined as patients with severe plasma leakage with shock and/or fluid accumulation with respiratory distress, severe bleeding, or severe organ impairment.

Considering the population and comorbidity distributions in Taiwan, the following underlying comorbidities and conditions were selected as important parameters for further model analysis: characteristics such as age 65 years or older; conditions such as DM, chronic kidney disease (CKD), or end-stage renal disease (ESRD), liver cirrhosis, chronic obstructive pulmonary disease (COPD), congestive heart failure (CHF), and neoplasms [22]; and clinical symptoms such as high or low temperature, tachypnea, tachycardia, and bleeding tendency as reflected in a prolonged aPTT, PT, and severe thrombocytopenia. Shock was defined as a systolic pressure < 90 mmHg with a diastolic blood pressure (DBP) < 60 mmHg and organ dysfunction. Tachycardia was defined as a heart rate > 120/min. Tachypnea was defined as a respiratory rate > 20/min. High fever was defined as a tympanic temperature ≥ 38.5 °C. Severe thrombocytopenia was defined as a platelet count < 100,000/µL. A prolonged aPTT was defined as international normalized ratio (INR) ≥ 1.2 and ≥50 s. Acute elevated liver enzymes were defined as an AST or ALT level ≥ 200 IU/mL. The International Classification of Diseases, 10th Revision, Clinical Modification (ICD-10-CM) ICD-10 codes used for chronic diseases were as follows: DM-E08-E10; asthma or COPD-J41, J43, J44, J45, J47; malignant neoplasms-C00-C90, D00-D49; CKD or ESRD-N059, N18; and chronic liver disease and cirrhosis-K740, K741, K742, K743, K744, K746, and CHF-I50. Dengue patients with critical outcomes were defined as those who progressed to death regardless of the presence of any warning signs or underlying diseases, and severe dengue was reported and confirmed by the Taiwan CDC. Severe dengue was defined by any of the following symptoms during the disease course: severe plasma leakage leading to shock or fluid accumulation with respiratory distress; severe bleeding; or severe organ impairment such as elevated transaminase levels ≥ 1000 IU/L, impaired consciousness, or heart impairment. Critical outcomes were determined by a consensus committee organized by the Taiwan CDC that included infectious disease and critical care specialists. The clinical epidemiologist analyzed the anonymous data and was unaware of the data collection process.

### 2.3. Model Analysis for the Dengue Severity Score

An efficient scoring system was created to identify critical outcomes in dengue patients based on basic clinical and laboratory features, including only those variables that are relevant and readily available when patients arrive at most hospitals. Previous literature reviews determined potential factors, e.g., vital signs and important laboratory data. Later, candidate factors were selected by multivariable binary logistic regression. The significant coefficients of parameters were transformed into a dengue severity score by inverse odds ratios [23]; this score was constructed by dividing each coefficient with the smallest coefficient (0.49) of the multivariate logistic regression models with backward stepwise selection. The predictor with the smallest coefficient is intuitive, considering the 1-point score mathematically presented. The severity scores were rounded up or down to the nearest 0.5.

### 2.4. Data Analysis

Potential indicators of critical outcomes in dengue patients were tested with a nonparametric test for trend. The Pearson’s chi-square test, the Mantel–Haenszel chi-square test, and logistic regression models were used to compare categorical and continuous variables between groups, yielding odds ratios (ORs) with 95% confidence intervals (CIs). The Mann–Whitney U test was used to compare continuous variables when the variable was either ordinal or continuous but not normally distributed. For all tests, a two-sided *p* ≤ 0.05 was considered statistically significant. Clinical vital signs, important and common laboratory data, and previously mentioned important outcome factors, such as thrombocytopenia (<100,000/μL), aPTT, were enrolled for the primary factor survey [24,25]. The predictive ability was analyzed by multivariable binary logistic regression and is presented as the coefficients and ORs. Potential factors for determining severity scores were derived by transforming the coefficients of the parameters and used to estimate the strength of the association [20,22,26,27,28]. Moreover, scores for predicting critical outcomes in dengue patients were calculated with receiver operating characteristic curve (ROC) analysis.

The optimal cutoff value was obtained by ROC curve analysis, and its sensitivity and specificity for predicting critical outcomes in dengue patients were measured. The area under the curve (AUC) defined how well the model could discriminate between critical and noncritical patients with dengue. In addition, dengue patients were dichotomized by the cutoff value obtained by generating an ROC curve and maximizing the Youden index (Youden index: J = max [sensitivity + specificity] − 1) [29]. The essential indicators, sensitivity, specificity, and likelihood ratios were analyzed to measure the diagnostic testing accuracy. SAS (Statistical Analysis Software 9.4, SAS Institute Inc., Cary, NC, USA) was used for all the data analyses.

### 2.5. Ethics Statement

The present study was approved by the Research Ethics Committee of the study medical centers. All the study protocols were performed in accordance with the principles of the Helsinki Declaration. Both the study protocol and the data were approved by the Institutional Review Board (IRB), National Health Research Institutes (EC1060705-E), National Cheng Kung University Hospital (A-ER-104-223) and Kaohsiung Municipal Siaogang Hospital (KMUHIRB-E(1)20200052). To protect personal privacy, patient information was collected and anonymized, and the patients were de-identified prior to analysis; therefore, the requirement for informed consent was waived by the IRB.

## 3. Results

During the dengue epidemic period, a total of 4229 dengue-confirmed adult patients visited the ED and OPD of the study hospital and were included in this study (Table S1). Table 1 demonstrates the significant differences in demographics between the dengue patients from the ED and OPD. Among the ED patients with confirmed dengue, 33.2% were elderly adults aged ≥65 years (Range: 18–103 years). Among the enrolled dengue patients with underlying comorbidities, the most common underlying diseases were DM (83, 12.4% in the ED; 8, 18.8% in the OPD), followed by neoplasms (60, 7.5% in the ED; 3, 9.3% in the OPD), CKD/ESRD (42, 6.4% in the ED; 5, 15.6% in the OPD), and liver cirrhosis (4, 0.6% in the ED; 0, 0% in the OPD). Compared with the patients who visited the OPD, patients who presented at the ED were relatively older and had a higher likelihood of comorbidities. A total of 4229 dengue patients were classified based on presentations at the first hospital visit as group A (may be sent home) of 1396 dengue patients and group B (referred for in-hospital care) of 2833 dengue patients according to the 2009 WHO criteria.

### 3.1. Major Parameters for Predicting Critical Outcomes in Dengue Patients

The dengue patients’ clinical conditions based on the WHO classification system are shown in Figure 1. Of the 4229 dengue patients, 3528 were excluded from prognostic tool development, 270 were initially diagnosed with severe dengue, and 3258 lacked complete vital signs and clinical data. The median duration of illness for these included and excluded participants visiting ED or OPD was one day (Table S2). Out of the 701 study subjects, 263 were diagnosed without warning signs and 438 were diagnosed with warning signs. We identified these patients according to the WHO management recommendations: 156 patients were classified into group A and 545 were classified into group B. In the course of treatment for dengue, 47 patients were later confirmed to have critical outcomes (Figure 1). A total of 701 cases with complete clinical and laboratory data were analyzed (Table 1).

The results of the chi-square tests or Fisher’s exact tests for the comparisons of demographic variables, clinical features, and laboratory data between the patients with and without critical outcomes in the study hospital who had complete clinical information are summarized in Table 2. The highest OR was 76.92 (95% CI: 18.45–322.58) for patients aged 65 years and older who had any warning signs (nausea, vomiting, hematuria, epistaxis, tarry stool, vaginal bleeding, abdominal pain, weakness, and poor appetite) or underlying diseases associated with poor dengue outcomes, including DM, CKD, ESRD, liver cirrhosis, COPD, CHF, and neoplasms [22,30,31].

Multiple parameters were integrated and analyzed by multivariate logistic regression analysis with backward selection, and the results are shown in Table 3. The smallest coefficient of the multivariate logistic regression models was 0.49 for patients with tachycardia. The estimated severity scores ranged from 1 to 4, and the sum of the total scores was 9. Significant parameters were used to construct possible scoring models. On the basis of the ORs and 95% CIs, dengue with critical outcomes was associated with patients with underlying disease (OR = 60.23, 95% CI: 14.11–257.05), a tympanic temperature lower than 38.5 °C (OR =2.68, 95% CI: 1.18–6.08), lower DBP (OR = 3.14, 95% CI: 1.01–9.76), aPTT ≥ 50 s (aOR = 5.76, 95% CI: 2.30–14.42), and elevated liver enzymes (OR = 3.16, 95% CI: 1.10–9.07).

The sensitivity, specificity, positive likelihood ratio (LR+), and negative likelihood ratio (LR−) from the ROC curve analysis are shown in Table 4. The sensitivities for predicting critical outcomes in dengue patients with scores of ≥1 and ≥9 were 100.00% and 4.26%, respectively. The LR+ values of 21.28, 23.19, and 97.40 for scores of 6, 7, and 8 show that the identification of patients with critical outcomes with those scores is robust. Alternatively, the LR− values of 0.03, 0.06, and 0.06 for scores of 2, 3, and 4 indicate that the ability of those scores to rule out dengue patients with critical outcomes is excellent.

**Figure 2:**
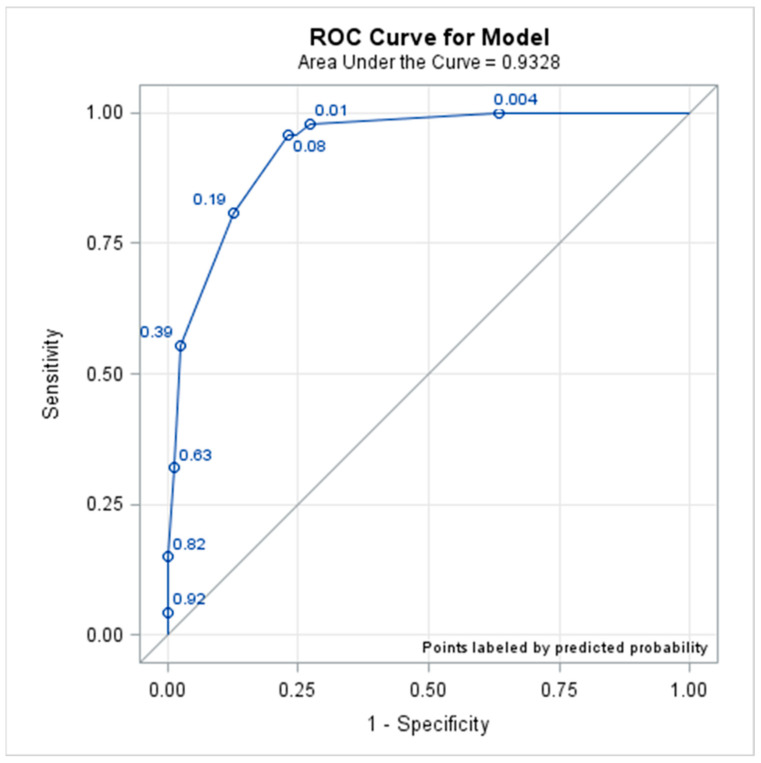
Receiver operating characteristic (ROC) curve for the logistic model with the sensitivity and specificity at the cutoff points for clinical and laboratory data.

ROC curve analysis was used to evaluate model performance (Figure 2). The AUC is equivalent to the probability that a randomly selected dengue patient with critical outcomes has a higher score than a randomly selected dengue patient without critical outcomes. This prognostic tool showed an AUC of 0.933 (95% CI: 0.905–0.960). The values of Youden’s index (sensitivity + specificity − 1) were also calculated to facilitate the selection of an appropriate cutoff value. The optimal probability cutoff is the maximum value of Youden’s index. The data show that the highest Youden’s index value of 0.725 was obtained with a cutoff score of 4. Overall, these findings confirmed the excellent performance of this model with regard to the identification of patients developing critical outcomes based on this dataset.

### 3.2. Utility Evaluation of the Indicators from the External Data

To further evaluate the utility of the indicators for identifying critical outcomes in dengue patients who presented dengue-associated symptoms/signs in the first three days, with the best cutoff value of 4, we enrolled 787 patients from another designated dengue hospital in Kaohsiung. The enrolled cases included dengue patients diagnosed from 2011 to 2015. A total of 65 out of 787 patients had critical outcome scores ≥4 (Table 5). The results for the dengue patients showed a high sensitivity (100%) and acceptable specificity (69%). The AUC of the ROC curve of 0.8 (0.768–0.832) with a Youden’s index value of 0.693 was within the acceptable range.

## 4. Discussion Estimation

In this study, we developed a new scoring system to predict critical outcomes in dengue patients who presented dengue-associated symptoms/signs in the first three days by integrating clinical presentations; age; chronic comorbidities, such as DM, CKD, chronic heart failure, and neoplasms; and abnormal laboratory findings. Given the importance of early identification and treatment of dengue with critical outcomes, the scoring algorithm proposed in this study can be considered clinically useful, especially during dengue outbreaks.

The surge in dengue patients resulted in an overload of the healthcare burden, which reveals the difficult problems inherent in early diagnosis of severe dengue and hospital beds allocation in the healthcare system. Our prediction tool provides primary physicians with a method of identifying patients likely to progress to dengue with critical outcomes early in the clinical course. Users can rapidly determine which patients with dengue infections may require further monitoring or intensive care simply using their age, warning signs, vital signs, and simple laboratory data, including aPTT and liver function results, to calculate their score. Dengue patients with severe disease can be treated successfully if they are diagnosed very early [10]. The development of a prognostic tool to detect the likelihood of progression to dengue with critical outcomes is warranted to reduce the morbidity and mortality rates associated with dengue [32]. The use of this scoring system could decrease unnecessary blood tests and reduce medical expenses in clinical settings. Early, rapid, and affordable prediction tools for dengue with critical outcomes may also limit unnecessary hospital admission, enable appropriate hospital bed allocation, and in turn decrease the burden on healthcare systems during dengue outbreaks. The present scoring system had a relatively low specificity for utility evaluation in the external data. Further studies that enroll more dengue patients are warranted to improve the performance of the scoring system.

This study generated regression models with backward selection to integrate the 2009 WHO dengue case classification system with laboratory data [10]. For a given cutoff value, a critical or noncritical classification can be made for each individual by comparing the measurement to the cutoff value, as was shown previously in a multiple regression model that was constructed and validated for the clinical diagnosis of dengue in the pediatric population [33]. In addition to sensitivity and specificity, our study provided another statistical tool, likelihood ratios (LRs), to understand diagnostic tests in clinical practice and to determine how much the utilization of a particular test will alter the probability. A positive likelihood ratio, or LR+, is the ratio between the probability of true positives and the probability of false positives. For example, an LR+ of 97.4 for a score of 8 means that the likelihood of dengue patients developing critical outcomes, known to be positive, is 97.4 compared to those who will not develop critical outcomes.

The AUC indicates the accuracy of the scoring system. The AUC shows how well the model can discriminate between critical and noncritical patients; AUCs between 0.90 and 1 indicate excellent performance. The AUC of 0.933 in our study suggests that the score we used in our study should be an effective measure of accuracy to discriminate dengue patients with critical and noncritical outcomes. The optimal Youden’s index value (0.725) is more intuitive for interpretation in the clinical setting than the metrics provided in previous studies. The cutoff value of 4 yielded the highest sensitivity (95.7%) and specificity (76.8%), with an LR+ of 4.1, which showed that the score had a strong discriminatory ability for predicting critical outcomes in dengue patients.

Compared to other predictive scores for critical dengue, this scoring system has several advantages. First, our data included the initial clinical presentations, inexpensive diagnostic laboratory data, and details of the underlying comorbidities. The broad scope of the factors included in this scoring system makes it better able than the well-known WHO classification system to predict dengue with critical outcomes. Although the WHO classification system considers underlying diseases and symptoms and suggests different management strategies, it does not mention detailed definitions of the underlying diseases. Second, our scoring system was developed on the basis of a population that included patients with mild cases who visited the OPD, patients with moderate cases who needed advanced care, and patients with severe cases who were admitted to ICUs. Approximately one-fifth of the total number of patients with dengue and half of the patients with severe dengue during this outbreak in Tainan city were enrolled in our study [22]. The broad coverage of the study population suggests that it is representative of the target population. Third, our study recruited a wide spectrum of patients with different risk factors for critical dengue. Approximately 40% of the enrolled population was of advanced age. These patients had relatively more comorbidities, such as diabetes, CKD, heart diseases, malignancies, and COPD, and the distributions of these comorbidities were similar to the comorbidity profiles in many middle- and high-income countries. Therefore, this scoring system should be generalizable for general use in most dengue-endemic or dengue-epidemic areas. The complexity of patients’ underlying conditions highlights the importance of the sensitivity of the scoring system for predicting the development of dengue with critical outcomes in this population. Early recognition of unstable vital signs, such as lower temperature, insufficient intravascular volume (low diastolic BP), and comorbidities, enables healthcare providers to promote the initiation of advanced care plans to improve clinical outcomes.

There are several limitations that need to be addressed. First, although nearly 80% of the screened and eligible patients were excluded due to missing data, the patients with missing data had mild clinical symptoms initially compared to the enrolled patients (Table S2). Excluding the patients with missing data would not interfere with the prognostic indicator analysis. The study hospital is designated as one of the major medical care centers for dengue fever by the Ministry of Health and Welfare, Taiwan. Nearly all dengue and severe dengue patients were included for analysis in our study during the study period. The study group should have enough representatives, and an evidence-based evaluation of the results should not be affected by missing data. Furthermore, the dengue patients diagnosed (regardless of whether they were admitted) at this hospital received OPD follow-ups in the same hospital. Dengue patients from other hospitals with critical conditions were also referred to the study hospital for further treatment. A retrospective cross-sectional study design was adopted that only measured the correlation of various indicators rather than causality. As a result, outcome misclassification was minimal. Second, the enrolled patients visited healthcare facilities in the first three days after disease onset. The initial presentations may have lacked sufficient information to predict critical outcomes in dengue patients. For these patients, optimal supportive care and follow-up were required. Our scoring system provided guidance regarding the need for monitoring. If a patient has a high score, admission to the ICU should be arranged immediately. Third, the majority of the patients included in the development and usefulness evaluation were infected with dengue virus (DENV) serotype 2 [34]. Whether this scoring system would be equally effective for patients infected or reinfected with other subtypes of dengue viruses, such as DENV3 and DENV4, needs to be confirmed. Fourth, the aim of this study was early identification of dengue patients at risk for critical outcomes. Our data only included clinical presentations in the first three days. It is warranted to evaluate whether the scoring system can be used for any day after the onset of the symptoms. Fifth, we could not differentiate whether the dengue patients had a primary or secondary infection because the serial paired characterization of types of antibody response is not routinely performed during the outbreak in Taiwan. However, few cases were infected in Tainan city with dengue infection before the severe epidemics in 2015, and thus the patients in this study were more likely to have primary infections [30,31]. Finally, the scoring system was developed in Taiwan, a country with universal healthcare and convenient access to healthcare facilities. Whether the scoring system is applicable to patients from different dengue-endemic countries still needs confirmation.

## 5. Conclusions

In conclusion, this scoring system based on clinical characteristics and inexpensive laboratory test results has good accuracy for predicting critical outcomes in dengue patients, with an AUC of 0.933. Our model had good predictive ability and was externally validated. More data are needed for reliability and validation testing.

## Figures and Tables

**Table 1:** Demographic characteristics of 701 enrolled dengue patients for model analysis.

Characteristic	ED (*n* = 669)	Outpatient Clinic (*n* = 32)	*p* Value
Age (years)			0.7124 ^a^
Mean (SD)	54.1 (19.2)	55 (19)	
Median (Q1, Q3)	56 (37, 71)	60 (39.5, 72)	
Temperature (°C)			0.0004 ^a^
Mean (SD)	38.3 (1)	37.6 (1)	
Median (Q1, Q3)	38.4 (37.5, 39)	37.2 (36.8, 38.3)	
Respiration rate (/min)			0.896 ^a^
Mean (SD)	19.7 (3.4)	19.8 (2.9)	
Median (Q1, Q3)	20 (18, 20)	20 (18, 20)	
Pulse (/min)			0.0018 ^a^
Mean (SD)	97.7 (19.3)	86.6 (18)	
Median (Q1, Q3)	97 (84, 111)	86 (71.5, 99.5)	
DBP (mmHg)			0.0006 ^a^
Mean (SD)	83 (15.9)	72.7 (14.9)	
Median (Q1, Q3)	82 (73, 91)	75 (61.5, 82)	
ALT (IU/L)			<0.0001 ^a^
Mean (SD)	51.4 (251.6)	183.7 (384.9)	
Median (Q1, Q3)	21 (12, 42)	41 (28, 102)	
AST (IU/L)			0.0036 ^a^
Mean (SD)	113.7 (609)	408 (1234.8)	
Median (Q1, Q3)	43 (30, 77)	82 (41, 141)	
Platelet count (10^3^/µL)			0.0001 ^a^
Mean (SD)	141.6 (69.8)	94.1 (858.3)	
Median (Q1, Q3)	140 (101, 184)	77 (26, 131.5)	
aPTT (sec)			0.0252 ^a^
Mean (SD)	39 (7)	44.8 (13.8)	
Median (Q1, Q3)	37.9 (34.8, 41.5)	41.8 (34.4, 52.6)	
Advanced age, *n* (%)			0.8889 ^b^
≥65 years	222 (33.2)	11 (34.4)	
<65 years	447 (66.8)	21 (65.6)	
Sex, *n* (%)			0.5695 ^b^
Male	348 (52.0)	15 (46.9)	
Female	321 (48.0)	17 (53.1)	
Comorbidities, *n* (%)			
DM	83 (12.4)	6 (18.8)	0.2788 ^c^
CKD/ESRD	42 (6.4)	5 (15.6)	0.0601 ^c^
Liver cirrhosis	4 (0.6)	0 (0)	1.0000 ^c^
Neoplasm	50 (7.5)	3 (9.3)	0.0005 ^c^
COPD	21 (3.1)	0 (0)	0.1944 ^c^
WHO classification			0.0006 ^b^
Group A	141 (21.1)	15 (46.9)	
Group B	528 (78.9)	17 (53.1)	

Q1: 25 percentile, Q3: 75 percentile; ^a^ Wilcoxon rank sum test (Mann-Whitney U test); ^b^ chi-square test; ^c^ Fisher’s exact test; CKD: chronic kidney disease; COPD: chronic obstructive pulmonary disease; DM: diabetes mellitus; ED: emergency department; ESRD: end-stage renal disease; SD: standard deviation.

**Table 2:** Characteristics of the analyzed dengue patients based on their critical outcomes.

Parameters	Critical Outcomes (*n* = 47)	Non-Critical Outcomes (*n* = 654)	OR (95% CI)	*p* Value
Group B *	45 (95.7)	500 (76.5)	76.92 (18.45–322.58)	<0.0001 ^a^
Male	23 (48.9)	340 (52)	0.89 (0.49–1.60)	0.6859 ^a^
Temperature ≥ 38.5 °C	10 (21.3)	329 (50.3)	0.27 (0.55–0.13)	0.0001 ^a^
Tachypnea ≥ 20/min	35 (74.5)	408 (62.4)	1.76 (0.90–3.45)	0.0971 ^a^
Tachycardia ≥ 120/min	6 (12.8)	85 (13)	0.98 (0.40–2.38)	0.9637 ^a^
DBP < 60 mmHg	8 (17)	23 (3.5)	5.63 (2.36–13.39)	0.0005 ^b^
Platelet count < 100,000/µL	29 (61.7)	154 (23.6)	5.23 (2.83–9.68)	<0.0001 ^a^
aPTT ≥ 50 s	17 (36.2)	24 (3.7)	14.88 (7.23–30.58)	<0.0001 ^b^
ALT or AST ≥ 200 U/L	12 (25.5)	29 (4.4)	7.39 (3.5–15.7)	<0.0001 ^b^

* Group B was characterized by any of the following: aged 65 years and older and any of the warning signs (nausea, vomiting, hematuria, epistaxis, tarry stool, vaginal bleeding, abdominal pain, weakness, and poor appetite) or dengue-related underlying diseases, including DM, CKD, ESRD, liver cirrhosis, COPD, CHF, and neoplasms. The reference group here is Group A; OR: odds ratio; ^a^ chi-square test; ^b^ Fisher’s exact test.

**Table 3:** Indicators for predicting critical outcomes in dengue patients in backward stepwise regression.

Characteristic	Odds Ratio * (95% CI)	*p*	Coefficient †	Score
Group B	60.23 (14.11–257.05)	<0.0001	2.05	4
Temperature < 38.5 °C	2.68 (1.18–6.08)	0.0183	0.49	1
Lower DBP	3.14 (1.01–9.76)	0.0479	0.57	1
Prolonged aPTT	5.76 (2.30–14.42)	0.0002	0.88	2
Elevated liver enzyme	3.16 (1.10–9.07)	0.0327	0.57	1

Group B was characterized by any of the following: aged 65 years and older and any of the warning signs (nausea, vomiting, hematuria, epistaxis, tarry stool, vaginal bleeding, abdominal pain, weakness, and poor appetite) or dengue-related underlying diseases, including DM, CKD, ESRD, liver cirrhosis, COPD, CHF, and neoplasms; * Estimate of intercept for the logistic regression model: −1.954; † The dengue severity score was constructed by dividing each coefficient with the smallest coefficient (0.49) of the multivariate logistic regression model.

**Table 4:** Criterion values and the associated sensitivity, specificity, positive likelihood ratio (LR+), and negative likelihood ratio (LR−) from the ROC curve analysis.

Criterion	Sensitivity (%) (95% CI)	Specificity (%) (95% CI)	LR+	LR−
≥1	100.00 (92.45–100.00)	36.39 (32.70–40.21)	1.57 (1.48–1.67)	—
≥2	97.87 (88.71–99.95)	72.63 (69.04–76.01)	3.58 (3.13–4.08)	0.03 (0–0.20)
≥3	95.74 (85.46–99.48)	75.38 (71.89–78.64)	3.89 (3.36–4.51)	0.06 (0.01–0.22)
≥4	95.74 (85.4–99.48)	76.76 (73.33–79.95)	4.12 (3.54–4.79)	0.06 (0.01–0.22)
≥5	80.85 (66.74–90.85)	87.46 (84.68–89.90)	6.45 (5.04–8.24)	0.22 (0.12–0.39)
≥6	55.32 (40.12–69.83)	97.40 (95.87–98.48)	21.28 (12.47–36.33)	0.46 (0.33–0.63)
≥7	31.91 (19.09–47.12)	98.62 (97.40–99.37)	23.19 (10.72–50.17)	0.69 (0.57–0.84)
≥8	14.89 (6.20–28.31)	99.85 (99.15–100.00)	97.40 (12.24–775.25)	0.85 (0.76–0.96)
≥9	4.26 (0.52–14.54)	100.00 (99.44–100.00)	—	0.96 (0.90–1.02)

LR+: sensitivity/(1 − specificity); LR: (1 − sensitivity)/specificity.

**Table 5:** Accuracy evaluation of the dengue critical scores from the external hospital.

Score Range	Critical Outcomes (*n* = 65)	Non-Critical Outcomes (*n* = 722)	Estimation		
			Sensitivity (%)	Specificity (%)	Accuracy *
Mean (SD)	5.2 (1)	3.1 (1.7)			
Median (Q1, Q3)	5 (5, 6)	2 (2, 5)			
Score ≥ 4	65	222	100.00 (94.48–100.00)	69.25 (65.74–72.60)	71.79 (68.51–74.91)

* Accuracy: Sensitivity × Prevalence + Specificity × (1 − Prevalence).

## Data Availability

The data are unavailable due to privacy or ethical restrictions. The data were obtained from National Cheng Kung University Hospital and are available with the permission of National Cheng Kung University Hospital.

## Supplementary Materials

The following supporting information can be downloaded at: https://www.mdpi.com/article/10.3390/tropicalmed8040188/s1. Table S1. Demographic characteristics of dengue patients in a teaching hospital during the 2015 dengue outbreak in Tainan, Taiwan; Table S2. Comparison of the demographic characteristics of included and excluded dengue patients in a teaching hospital during the 2015 dengue outbreak in Tainan, Taiwan.

## References

[1] Brady O.J., Gething P.W., Bhatt S., Messina J.P., Brownstein J.S., Hoen A.G., Moyes C.L., Farlow A.W., Scott T.W., Hay S.I. (2012). Refining the global spatial limits of dengue virus transmission by evidence-based consensus. PLoS Negl. Trop. Dis..

[2] Guzman M.G., Halstead S.B., Artsob H., Buchy P., Farrar J., Gubler D.J., Hunsperger E., Kroeger A., Margolis H.S., Martinez E. (2010). Dengue: A continuing global threat. Nat. Rev. Microbiol..

[3] Lee M.S., Hwang K.P., Chen T.C., Lu P.L., Chen T.P. (2006). Clinical characteristics of dengue and dengue hemorrhagic fever in a medical center of southern Taiwan during the 2002 epidemic. J. Microbiol. Immunol. Infect..

[4] CDC Annual Report 2016. https://www.cdc.gov.tw/InfectionReport/Info/MBykt7DHDDcSbrcfxoLRoQ?infoId=HCzh6FgAgovrYiZVrpJrbw.

[5] Hsu J.C., Hsieh C.L., Lu C.Y. (2017). Trend and geographic analysis of the prevalence of dengue in Taiwan, 2010-2015. Int. J. Infect. Dis..

[6] Mohd-Zaki A.H., Brett J., Ismail E., L’Azou M. (2014). Epidemiology of dengue disease in Malaysia (2000–2012): A systematic literature review. PLoS Negl. Trop. Dis..

[7] Bravo L., Roque V.G., Brett J., Dizon R., L’Azou M. (2014). Epidemiology of dengue disease in the Philippines (2000–2011): A systematic literature review. PLoS Negl. Trop. Dis..

[8] Anders K.L., Nguyet N.M., Chau N.V., Hung N.T., Thuy T.T., Lien Le B., Farrar J., Wills B., Hien T.T., Simmons C.P. (2011). Epidemiological factors associated with dengue shock syndrome and mortality in hospitalized dengue patients in Ho Chi Minh City, Vietnam. Am. J. Trop. Med. Hyg..

[9] Rigau-Perez J.G., Clark G.G., Gubler D.J., Reiter P., Sanders E.J., Vorndam A.V. (1998). Dengue and dengue haemorrhagic fever. Lancet.

[10] World Health Organization Dengue: Guidelines for Diagnosis, Treatment, Prevention and Control: New Edition. http://www.ncbi.nlm.nih.gov/pubmed/23762963.

[11] Pang J., Thein T.L., Leo Y.S., Lye D.C. (2014). Early clinical and laboratory risk factors of intensive care unit requirement during 2004-2008 dengue epidemics in Singapore: A matched case-control study. BMC Infect. Dis..

[12] Low J.G., Ong A., Tan L.K., Chaterji S., Chow A., Lim W.Y., Lee K.W., Chua R., Chua C.R., Tan S.W. (2011). The early clinical features of dengue in adults: Challenges for early clinical diagnosis. PLoS Negl. Trop. Dis..

[13] Pang J., Leo Y.S., Lye D.C. (2016). Critical care for dengue in adult patients: An overview of current knowledge and future challenges. Curr. Opin. Crit. Care.

[14] Liew S.M., Khoo E.M., Ho B.K., Lee Y.K., Omar M., Ayadurai V., Mohamed Yusoff F., Suli Z., Mudin R.N., Goh P.P. (2016). Dengue in Malaysia: Factors Associated with Dengue Mortality from a National Registry. PLoS ONE.

[15] Pinto R.C., Castro D.B., Albuquerque B.C., Sampaio Vde S., Passos R.A., Costa C.F., Sadahiro M., Braga J.U. (2016). Mortality Predictors in Patients with Severe Dengue in the State of Amazonas, Brazil. PLoS ONE.

[16] Thein T.L., Leo Y.S., Fisher D.A., Low J.G., Oh H.M., Gan V.C., Wong J.G., Lye D.C. (2013). Risk factors for fatality among confirmed adult dengue inpatients in Singapore: A matched case-control study. PLoS ONE.

[17] Gubler D.J. (1998). Dengue and dengue hemorrhagic fever. Clin. Microbiol. Rev..

[18] Guzman M.G., Kouri G. (2002). Dengue: An update. Lancet. Infect. Dis..

[19] Chien Y.W., Wang C.C., Wang Y.P., Lee C.Y., Perng G.C. (2020). Risk of Leukemia after Dengue Virus Infection: A Population-Based Cohort Study. Cancer Epidemiol. Biomark. Prev..

[20] Yeh C.Y., Chen P.L., Chuang K.T., Shu Y.C., Chien Y.W., Perng G.C., Ko W.C., Ko N.Y. (2017). Symptoms associated with adverse dengue fever prognoses at the time of reporting in the 2015 dengue outbreak in Taiwan. PLoS Negl. Trop. Dis..

[21] Diagnostics S. SD BIOLINE Dengue Duo. https://www.globalpointofcare.abbott/en/product-details/sd-bioline-dengue-duo-ns1-ag---ab-combo.html.

[22] Hsieh C.C., Cia C.T., Lee J.C., Sung J.M., Lee N.Y., Chen P.L., Kuo T.H., Chao J.Y., Ko W.C. (2017). A Cohort Study of Adult Patients with Severe Dengue in Taiwanese Intensive Care Units: The Elderly and APTT Prolongation Matter for Prognosis. PLoS Negl. Trop. Dis..

[23] Pongpan S., Wisitwong A., Tawichasri C., Patumanond J., Namwongprom S. (2013). Development of dengue infection severity score. ISRN Pediatr..

[24] Vijayaraghavan, Thay Wee Y., Foong Shing W., Hafsa P. (2020). Predictors of Dengue Shock Syndrome: APTT Elevation as a Risk Factor in Children with Dengue Fever. J. Infect. Dis. Epidemiol..

[25] Hamsa B.T., Srinivasa S.V., Prabhakar K., Raveesha A., Manoj A.G. (2019). Significance of APTT as early predictor of bleeding in comparison to thrombocytopenia in dengue virus infection. Int. J. Res. Med. Sci..

[26] Kuo H.J., Lee I.K., Liu J.W. (2018). Analyses of clinical and laboratory characteristics of dengue adults at their hospital presentations based on the World Health Organization clinical-phase framework: Emphasizing risk of severe dengue in the elderly. J. Microbiol. Immunol. Infect..

[27] Werneck G.L., Macias A.E., Mascarenas C., Coudeville L., Morley D., Recamier V., Guergova-Kuras M., Puentes-Rosas E., Baurin N., Toh M.L. (2018). Comorbidities increase in-hospital mortality in dengue patients in Brazil. Mem. Do Inst. Oswaldo Cruz.

[28] Sangkaew S., Ming D., Boonyasiri A., Honeyford K., Kalayanarooj S., Yacoub S., Dorigatti I., Holmes A. (2021). Risk predictors of progression to severe disease during the febrile phase of dengue: A systematic review and meta-analysis. Lancet. Infect. Dis..

[29] Gönen M. (2010). A Review of: “ROC Curves for Continuous Data, by W. J. Krzanowski and D. J. Hand”. J. Biopharm. Stat..

[30] Chien Y.W., Chuang H.N., Wang Y.P., Perng G.C., Chi C.Y., Shih H.I. (2022). Short-term, medium-term, and long-term risks of nonvariceal upper gastrointestinal bleeding after dengue virus infection. PLoS Negl. Trop. Dis..

[31] Chien Y.W., Huang H.M., Ho T.C., Tseng F.C., Ko N.Y., Ko W.C., Perng G.C. (2019). Seroepidemiology of dengue virus infection among adults during the ending phase of a severe dengue epidemic in southern Taiwan, 2015. BMC Infect. Dis..

[32] Wong P.F., Wong L.P., AbuBakar S. (2020). Diagnosis of severe dengue: Challenges, needs and opportunities. J. Infect. Public Health.

[33] Diaz-Quijano F.A., Figueiredo G.M., Waldman E.A., Figueiredo W.M., Cardoso M.R.A., Campos S.R.C., Costa A.A., Pannuti C.S., Luna E.J.A. (2019). Comparison of clinical tools for dengue diagnosis in a pediatric population-based cohort. Trans. R Soc. Trop. Med. Hyg..

[34] Wang S.F., Chang K., Loh E.W., Wang W.H., Tseng S.P., Lu P.L., Chen Y.H., Chen Y.A. (2016). Consecutive large dengue outbreaks in Taiwan in 2014–2015. Emerg. Microbes. Infect..

