# Supplementary material for: Development and Utility of Practical Indicators of Critical Outcomes in Dengue Patients Presenting to Hospital: A Retrospective Cross-Sectional Study
Source: Trop Med Infect Dis. 2023 Mar 25;8(4):188. doi: 10.3390/tropicalmed8040188 (PMC10142425; doi:10.3390/tropicalmed8040188)
Supplement: Supplementary file 1 [file tropicalmed-08-00188-s001.zip › tropicalmed-2189234-supplementary.pdf]

Table S1. Demographic characteristics of dengue patients in a teaching hospital during the 2015 dengue outbreak in Tainan, Taiwan.

Characteristic	ED (n=3490)	OPD (n=739)	p value
Age (years)			
Mean (SD)	49.7 (22)	43.3 (19.4)	<0.0001 [a]
Median (Q1, Q3)	53 (30, 68)	42 (26, 60)	
Temperature (°C)			
Mean (SD)	38.3 (1)	37.9 (1)	<0.0001 [a]
Median (Q1, Q3)	38.5 (37.7, 39)	37.8 (37.2, 38.7)	
Respiration rate (/min)			
Mean (SD)	19.7 (3)	19.8 (3.6)	0.0703 [a]
Median (Q1, Q3)	20 (18, 20)	19 (18, 20)	
Pulse (/min)			
Mean (SD)	99.9 (19.2)	90.1 (22.6)	<0.0001 [a]
Median (Q1, Q3)	100 (86, 113)	88.5 (78, 100)	
DBP (mmHg)			
Mean (SD)	81.9 (15.5)	72.7 (13.4)	<0.0001 [a]
Median (Q1, Q3)	81 (72, 90)	72 (64, 80)	
ALT (IU/L)			
Mean (SD)	39.5 (135)	54 (155.7)	<0.0001 [a]
Median (Q1, Q3)	19 (10, 39)	24 (12, 49)	
AST (IU/L)			
Mean (SD)	91.7 (400.2)	89.8 (271.3)	0.7901 [a]
Median (Q1, Q3)	43 (31, 71)	44 (32, 70)	
Platelet count (103/μL)			
Mean (SD)	148.8 (64.8)	144.2 (65.0)	0.0482 [a]
Median (Q1, Q3)	148 (108, 189)	144 (102, 186)	
aPTT (sec)			
Mean (SD)	39.4 (8.6)	39.9 (8.2)	0.0311 [a]
Median (Q1, Q3)	38 (34.9, 41.9)	39 (35.8, 41.9)	
Advanced age, N (%)			
≥65 years	1051 (30.1)	120 (16.2)	<0.0001 [b]
<65 years	2439 (69.9)	619 (83.8)	
Sex, N (%)			
Male	1753 (50.2)	398 (53.9)	0.0732 [b]
Female	1737 (49.8)	341 (46.1)	
Comorbidities, N (%)			
DM	405 (11.6)	45 (6.1)	<0.0001 [b]
CKD/ESRD	163 (4.7)	22 (3)	0.0409 [b]
Liver cirrhosis	23 (0.7)	2 (0.3)	0.2392 [c]

Neoplasm	207 (5.9)	37 (5)	<0.0001 [c]
COPD	79 (2.4)	0 (0)	<0.0001 [c]
WHO classification			<0.0001[b]
Group A	814 (23.3)	582 (78.8)	
Group B	2676 (76.7)	157 (21.2)	

Q1: 25 percentile, Q3: 75 percentile; a Wilcoxon rank sum test (Mann-Whitney U test); b Chi-square test; c Fisher's exact test; CKD: chronic kidney disease; COPD: chronic obstructive pulmonary disease; DM: diabetes mellitus; ED: emergency department; ESRD: end-stage renal disease; SD: standard deviation.

Table S2. Comparison of the demographic characteristics of included and excluded dengue patients in a teaching hospital during the 2015 dengue outbreak in Tainan, Taiwan

**Characteristic**	**Included (n=701)**	**Excluded (n=3528)**	**p value**
Age (years)			
Mean (SD)	61.5 (16.5)	46.0 (21.7)	<0.0001[a]
Median (Q1, Q3)	53 (52, 75)	46 (27, 64)	
Length of illness (days)			
Mean (SD)	1 (1.2)	1 (1.1)	0.7475[a]
Median (Q1, Q3)	1 (0, 2)	1 (0, 2)	
Temperature (°C)			
Mean (SD)	38.2 (1.0)	38.3 (1.0)	0.2971[a]
Median (Q1, Q3)	38.3 (37.5, 39)	38.4 (37.6, 39)	
Respiration rate (/min)			
Mean (SD)	19.7 (2.6)	19.7 (3.0)	0.8429[a]
Median (Q1, Q3)	20 (18, 20)	20 (18, 20)	
Pulse (/min)			
Mean (SD)	95.7 (18.7)	99.7 (19.9)	<0.0001[a]
Median (Q1, Q3)	95 (83, 109)	100 (85, 113)	
DBP (mmHg)			
Mean (SD)	81.4 (15.6)	80.4 (15.5)	0.2096[a]
Median (Q1, Q3)	80 (71, 91)	80 (71, 89)	
ALT (IU/L)			
Mean (SD)	51.6 (258.2)	40.4 (102.2)	0.0003[a]
Median (Q1, Q3)	23 (13, 45)	19 (10, 39)	
AST (IU/L)			
Mean (SD)	124.4 (667.6)	89.6 (369.7)	0.0002[a]

Median (Q1, Q3)	49 (33, 86)	43 (31, 68)	
Platelet count (10^3/μL)			
Mean (SD)	136.0 (65.6)	150.3 (64.5)	<0.0001[a]
Median (Q1, Q3)	138.5 (91, 177)	148.5 (111, 190)	
aPTT (sec)			
Mean (SD)	40.1 (11.6)	39.5 (7.7)	0.905[a]
Median (Q1, Q3)	38.1 (34.7, 43)	38.4 (35.2, 41.8)	
Advanced age, N (%)			
≥65 years	315 (44.9)	856 (24.3)	<0.0001[b]
<65 years	386 (55.1)	2672 (75.7)	
Sex, N (%)			
Male	323 (46.1)	1828 (51.8)	0.0055[b]
Female	378 (53.9)	1700 (48.2)	
Comorbidities, N (%)			
DM	127 (18.1)	323 (9.2)	<0.0001[b]
CKD/ESRD	56 (8.0)	129 (3.7)	<0.0001[b]
Liver cirrhosis	11 (1.6)	14 (0.4)	0.0011[c]
Neoplasm	66 (10.9)	178 (6.1)	<0.0001[c]
COPD	12 (1.7)	67 (1.9)	0.0738[c]
WHO classification			<0.0001[b]
Group A	156 (22.2)	1246 (35.3)	
Group B	545 (77.8)	2282 (64.7)	

Q1: 25 percentile, Q3: 75 percentile. [a] Wilcoxon rank sum test (Mann-Whitney U test). [b] Chi-square test. [c] Fisher's exact test. CKD: chronic kidney disease; COPD: chronic obstructive pulmonary disease; DM: diabetes mellitus; ED: emergency department; ESRD: end-stage renal disease; SD: standard deviation.
